# Advancing Risk Analysis of COVID-19 Clinical Predictors: The Case of Fasting Blood Glucose

**DOI:** 10.3389/fmed.2021.636065

**Published:** 2021-04-09

**Authors:** Hamad Ali, Abdullah A. Al-Shammari, Barrak Alahmad, Fahd Al-Mulla

**Affiliations:** ^1^Department of Medical Laboratory Sciences, Faculty of Allied Health Sciences, Health Sciences Center, Kuwait University, Kuwait City, Kuwait; ^2^Department of Genetics and Bioinformatics, Dasman Diabetes Institute (DDI), Kuwait City, Kuwait; ^3^Department of Mathematics, Faculty of Sciences, Kuwait University, Kuwait City, Kuwait; ^4^Department of Environmental Health, Harvard T.H. Chan School of Public Health, Harvard University, Boston, MA, United States

**Keywords:** COVID-19, risk analysis, nonlinearity, glucose, clinical outcomes, diabetes, glycolysis, inflammation

## Highlights

- Many of the clinical predictors of COVID-19 are naturally continuous.
- Such continuity may imply that a complex predictor-risk relationship is underplay.
- Risk analyses that allow continuous predictors to take a restriction-free shape can provide a better understanding of the clinical course of the disease. This refined approach can help generate hypotheses characterizing the mechanisms of disease progression.

To understand or predict the effects of serum glucose on COVID-19 outcomes such as hospitalization, intensive care unit (ICU) admission or death, one could try to use conventional regression techniques with glucose as the independent variable and one of these outcomes as the dependent variable. But how should the glucose variable be included in such models? One may try applying clinical threshold values to fit the regression model. For example, in the context of diabetes diagnosis, we can use the threshold values of hemoglobin A1c (A1C); A1C <5.7%, A1C between 5.7 and 6.4%, and A1C ≥6.5% to characterize patients as normal, prediabetic, or diabetic, respectively (1). Alternatively, we can use two categories instead of three: diabetics vs. non-diabetics. These threshold or categorical approaches, albeit commonly useful for identifying high risk groups, have underlying limitations. First, they assume complete homogeneity within each group, hence patients with A1C values of 6.5 and 10% are to be considered clinically identical. Put another way, these approaches assume that patients with A1C of 5.69% are entirely different from those with A1C of 5.70%. Secondly, the dose-response relationship is a step or staircase function, which is rarely a realistic description of real-life patient risks (2, 3).

**Figure 1:**
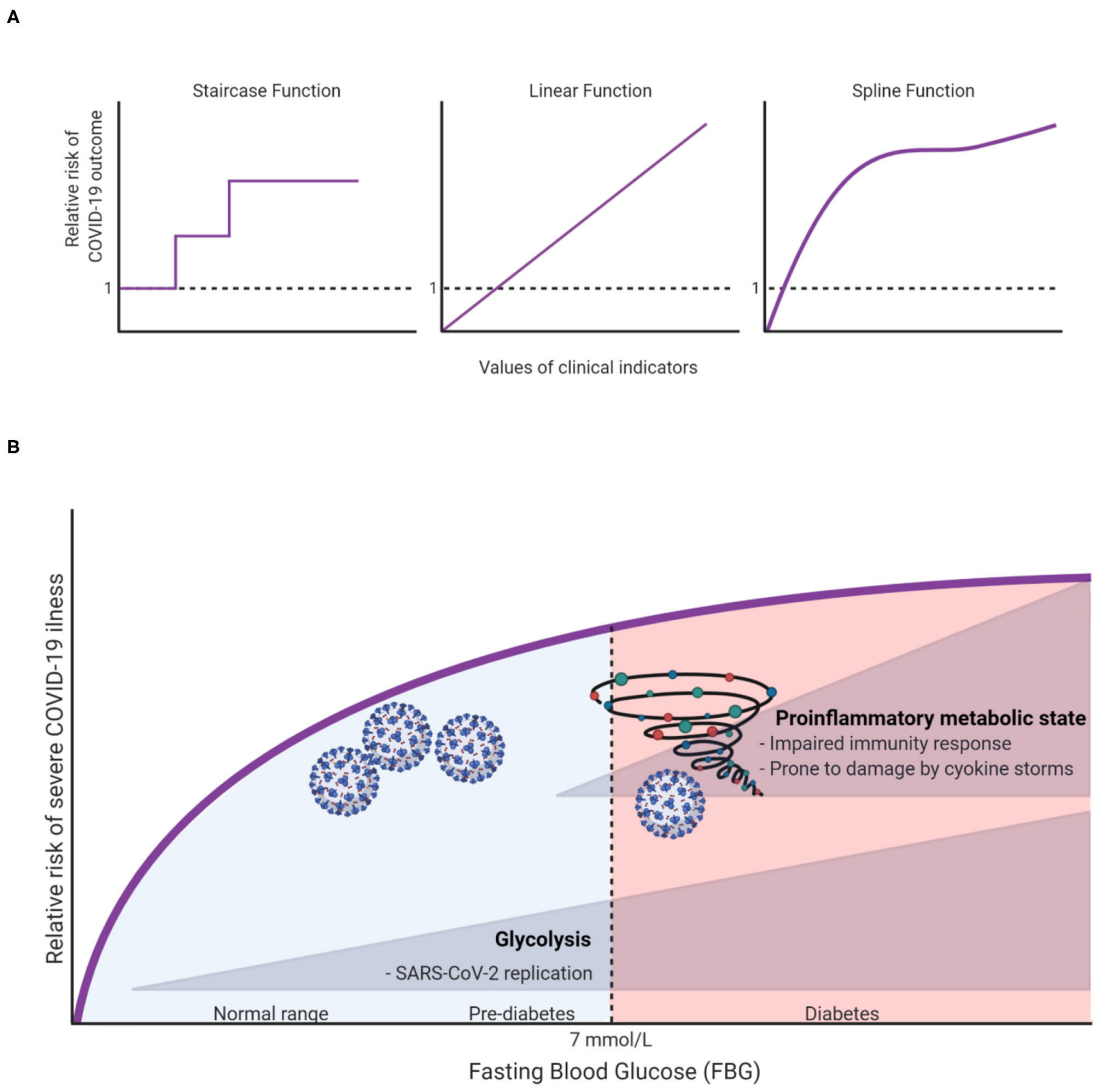
**(A)** Illustration of different possible dose-response relationships that can be fitted for continuous clinical indicators (e.g., age, fasting blood glucose, body mass index, etc.) to show the risk ratios of COVID-19 outcomes (e.g., hospitalization, ICU admission, or death). **(B)** A hypothesis on how FBG can affect COVID19 outcomes.

To capture the natural trends of a continuous exposure variable, one may surprisingly benefit from allowing the dose-response relationship to take whatever natural shape the data describe, rather than forcing it to fit idealized relationships such as linear (straight line) and categorical (staircase) functions (Figure 1A). The risk analyses based on such natural relationships are only made possible with modern computational algorithms. Take penalized splines as an example. These are smoothing non-parametric functions that, unlike forcing steps and lines, allow significant flexibility in estimating the dose-response curve. The only thing that governs these specific types of splines is, in fact, the goodness-of-fit. In other words, this smoothing of the relationship comes without idealized assumptions and prevents under- or over-fitting the data (4).

To illustrate, let us take fasting blood glucose (FBG) as an indicator for in-hospital complications among COVID-19 patients. Creating a three diagnostic categories of FBG [<6.1 mmol/L (reference), 6.1–6.9 mmol/L and ≥7.0 mmol/L] demonstrated that the odds ratios (OR) of developing 28-day in-hospital complications for the higher categories were 3.99 and 2.61, respectively (5). However, it remains unclear how much risk is associated with increasing FBG within the range of each group, and whether the patients within each group have a sufficiently homogeneous risk. Applying splines for the glucose variable suggested that even small changes in FBG within normal ranges can significantly increase the risk of severe illness (6). Surely this cannot be overlooked clinically, hence warranting recommendations for strict monitoring of FBG upon admission. Unexpectedly, this “unconventional” type of risk analysis has also brought to light an important and uncharted scientific question: why and how glucose can influence the outcome of COVID-19 even within normal ranges? At this stage we can only speculate as to what the answer might be, but we hope to inspire further research into this subject. In this spirit, we argue that there are potentially two independent mechanisms in which glucose can influence COVID-19 outcomes. First, at high levels of glucose (in the diabetic ranges), low-grade chronic inflammation state disturbs the homeostatic glucose regulation and insulin sensitivity. This could also in turn disrupt normal immune response by weakening T-cell function and add to the risk of hyperinflammation and cytokine storm syndrome which is associated with worse COVID-19 outcome (7). On the other hand, increases of FBG, even within normal ranges could affect COVID-19 outcomes through enhancing aerobic glycolysis in the infected monocytes with SARS-CoV2 which in turn enhance and facilitate viral replication and infection resulting in more severe outcome (8). The inflammation and glycolysis mechanisms are likely to be affected by different levels of FBG with the latter being sensitive to lower levels (Figure 1B).

The novelty presented here is the application of well-known tools that are not being applied much in the COVID-19 epidemiology, because in many cases, researchers opt to conventional and clinically straightforward approaches such as linear, dichotomous and categorical modeling. While this was acceptable for some time because of the computational complexity of the smoothing applications, they can be easily implemented in modern time computers and statistical softwares. We argue that they ought to be used.

Although utilizing smoothing functions, in our case, sounds reasonable, we must always exercise caution with smaller sample sizes. In addition, relying on cross-validation to determine penalty terms for penalized splines is computationally extensive. For example, the leave-one-out validation will leave one observation out at a time; fit the model on the remaining training data; test on the held-out data point and so on. An alternative approach to specify penalized splines is using Restricted Maximum Likelihood, which is a Likelihood based approach. Furthermore, interpretation of coefficients is not straightforward. Improving the fit of the dose-response relationship comes at the expense of easy interpretation.

What we are advocating for in this opinion piece is the mere attention to the nature of the dose-response relationship which is usually overlooked by simplifying assumptions such as forcing a straight line or forcing a staircase shape. In fact, with the pandemic hitting us harder, we need to leverage all the tools we have in the toolbox in order to get a better understanding of the complex pathophysiology of clinical predictors (like FBG) during the state of infection.

Bottom line, non-linearities, steep slopes, plateaus, or any other shape should always be considered for continuous variables such as serum blood glucose or A1C, perhaps even age, body mass index, and so on. In the age of big data, electronic health records, and artificial intelligence the conventional practices maybe too archaic. Once we correctly characterize these complex relationships, we can better capture the clinical course of the disease.

## Author Contributions

All authors listed have made a substantial, direct and intellectual contribution to the work, and approved it for publication.

## Conflict of Interest

The authors declare that the research was conducted in the absence of any commercial or financial relationships that could be construed as a potential conflict of interest.

## References

[1] American Diabetes Association. 2. Classification and diagnosis of diabetes: standards of medical care in diabetes-2020. Diabetes Care. (2020) 43:S14–31. 10.2337/dc20-S002 31862745

[2] Yoshimura I. The effect of measurement error on the dose-response curve. Environ. Health Perspect. (1990) 87:173–8. 2269223 10.1289/ehp.9087173 PMC1567852

[3] National Research Council. Chapter 12: Issues in the Assessment of Dose Response. In: Assessing the Human Health Risks of Trichloroethylene: Key Scientific Issues. Washington, DC: National Academies Press (2007).

[4] Wood SN Pya N Säfken B. Smoothing parameter and model selection for general smooth models. J Am Stat Assoc. (2016) 111:1548–63. 10.1080/01621459.2016.1180986

[5] Wang S Ma P Zhang S Song S Wang Z Ma Y. Fasting blood glucose at admission is an independent predictor for 28-day mortality in patients with COVID-19 without previous diagnosis of diabetes: a multi-centre retrospective study. Diabetologia. (2020) 63:2102–11. 10.1007/s00125-020-05209-1 32647915 PMC7347402

[6] Alahmad B Al-Shammari AA Bennakhi A Al-Mulla F Ali H. Fasting blood glucose and COVID-19 severity: nonlinearity matters. Diabetes Care. (2020) 43:3113–6. 10.2337/dc20-1941 33051331 PMC7770269

[7] Apicella M Campopiano MC Mantuano M Mazoni L Coppelli A Del Prato S. COVID-19 in people with diabetes: understanding the reasons for worse outcomes. Lancet Diabetes Endocrinol. (2020) 8:782–92. 10.1016/S2213-8587(20)30238-2 32687793 PMC7367664

[8] Codo AC Davanzo GG de Brito Monteiro L de Souza GF Muraro P Virgilio-da-Silva JV. Elevated glucose levels favor Sars-Cov-2 infection and monocyte response through a Hif-1α/Glycolysis dependent axis. Cell Metab. (2020) 3:437–46. 10.1016/j.cmet.2020.07.007 32697943 PMC7367032

